# Relationship between neuroticism, childhood trauma and cognitive-affective responses to auditory verbal hallucinations

**DOI:** 10.1038/srep34401

**Published:** 2016-10-04

**Authors:** Suzanne Ho-wai So, Marieke J. H. Begemann, Xianmin Gong, Iris E. Sommer

**Affiliations:** 1 Department of Psychology, The Chinese University of Hong Kong, Hong Kong SAR, China; 2 Department of Psychiatry, University Medical Center Utrecht, The Netherlands

## Abstract

Neuroticism has been shown to adversely influence the development and outcome of psychosis. However, how this personality trait associates with the individual’s responses to psychotic symptoms is less well known. Auditory verbal hallucinations (AVHs) have been reported by patients with psychosis and non-clinical individuals. There is evidence that voice-hearers who are more distressed by and resistant against the voices, as well as those who appraise the voices as malevolent and powerful, have poorer outcome. This study aimed to examine the mechanistic association of neuroticism with the cognitive-affective reactions to AVH. We assessed 40 psychotic patients experiencing frequent AVHs, 135 non-clinical participants experiencing frequent AVHs, and 126 healthy individuals. In both clinical and non-clinical voice-hearers alike, a higher level of neuroticism was associated with more distress and behavioral resistance in response to AVHs, as well as a stronger tendency to perceive voices as malevolent and powerful. Neuroticism fully mediated the found associations between childhood trauma and the individuals’ cognitive-affective reactions to voices. Our results supported the role of neurotic personality in shaping maladaptive reactions to voices. Neuroticism may also serve as a putative mechanism linking childhood trauma and psychological reactions to voices. Implications for psychological models of hallucinations are discussed.

Neuroticism is a personality trait characterized by emotional instability and proneness to experiencing anxiety, fear, and sadness^1^,^2^. The association between neuroticism and mood disorders such as major depression and generalized anxiety disorder has been well established^3^,^4^,^5^,^6^. More recently, there has been an increase in evidence linking neuroticism with psychosis. Wiltink and colleagues reported a significant association between neuroticism and perceptual abnormalities in non-clinical adolescents, even after taking depression into account^7^. Epidemiological data revealed that individuals with neuroticism are at increased risk of subsequently developing psychosis. This prediction remained significant even after adjusting for levels of anxiety and depression, supporting the argument for neuroticism as one of the vulnerability factors for psychosis^8^,^9^,^10^. Lysaker and colleagues found that patients with schizophrenia spectrum disorders who were high on neuroticism tended to have more severe positive symptoms, and to adopt an avoidant coping style when under stress^11^,^12^.

Although there is rich evidence that neuroticism contributes to the development and outcome of psychosis in both clinical and non-clinical groups, how this personality trait associates with the individual’s responses to specific psychotic symptoms is less well known. Auditory verbal hallucination (AVH) refers to the phenomenon where individuals report hearing voices, with a sufficient sense of reality, but without the presence of corresponding external stimulation^13^. These sensory experiences have been reported in patients with psychotic disorders, as well as other psychiatric disorders such as borderline personality disorder, bipolar disorder and severe mood disorders^14^,^15^. AVHs also occur in people without a psychiatric diagnosis, at a median prevalence rate of 13.4%^16^. Phenomenological studies have revealed similarities in physical characteristics of AVHs across clinical and non-clinical groups, such as the perceived location of the voices, volume, and number of voices^17^. However, there are important differences in both phenomenological characteristics and the individuals’ reactions, too. Patients are more likely to attribute their voices to specific people or agencies, and to appraise voices as uncontrollable, malevolent, powerful and dangerous^17^,^18^,^19^,^20^. Patients are also typically more distressed by the voices than non-clinical voice hearers^15^,^17^,^19^. The present study aimed at examining the mechanistic association of neuroticism with the cognitive-affective reactions to AVH in clinical and non-clinical voice hearers.

In noise stress research, individuals high on neuroticism have been shown to be more emotionally aroused in noisy and stressful conditions, manifesting more anxiety and worry^21^. When asked to perform mental tasks under a noisy condition, their performance was also more severely impacted than in non-neurotic individuals^21^. This suggests that neuroticism enhances affective reactivity to stressors. Therefore, we hypothesized that neuroticism would predict a higher level of emotional distress and resistance in response to AVH. Furthermore, according to the cognitive model proposed by Birchwood and Chadwick^22^,^23^, hallucinatory distress and behavioral resistance against voices is mediated by appraisal of voices as malevolent and powerful^24^. Since neuroticism is also characterized by a pervasive perception that the world is dangerous and threatening, and that one is unable to manage in face of challenges^25^,^26^,^27^, our second hypothesis was that neuroticism would predict a stronger tendency to appraise AVHs as malevolent and powerful.

Contrary to the historical view that neuroticism is fixed and genetically based, evidence of gene-environment interactions has raised the possibility that environmental factors, such as childhood trauma, may underlie the development of neuroticism^28^,^29^,^30^. It has been suggested that repeated exposure to aversive experiences may sensitize the individual into becoming more emotionally reactive^31^,^32^,^33^, purportedly by inducing a sense of unpredictability and uncontrollability^26^,^27^,^34^. In patients with schizophrenia, Lysaker found that those who were exposed to childhood sexual abuse exhibited significantly higher levels of neuroticism than those who did not have a history of abuse^35^. According to Birchwood *et al*.^22^, and Kilcommons and Morrison’s^36^ cognitive models of AVH, traumatic experiences may render an individual more vulnerable to negative schemas about the self, others, and the world, which may in turn influence his/her appraisals of AVH. In summary, childhood trauma has been shown to be associated with both neuroticism and cognitive-affective reactions to AVH. There is ample evidence supporting an association of childhood trauma with occurrence of psychosis^37^,^38^,^39^,^40^ and AVH^41^,^42^,^43^,^44^,^45^. However, whether neuroticism mediates the link between childhood trauma and an individual’s cognitive-affective reactions to AVH has not been tested directly. As emotional responses to and beliefs about voices are typical targets of cognitive behavior therapy for psychosis, investigating the factors that maintain these reactions to AVH bears clinical importance.

In the current study, we will test whether there is an association in both clinical and non-clinical voice hearers, between level of neuroticism and level of emotional distress in response to AVH. We will also test the association between level of neuroticism and levels of perceived power and malevolence of AVH, as well as resistance against AVH. Finally, we will test if the level of neuroticism mediates the associations between childhood trauma and these cognitive-affective reactions to voices.

## Methods

Research ethics for all methods involved in this study was approved by the Human Ethics Committee of the University Medical Center Utrecht. The authors assert that all procedures contributing to this work comply with the ethical standards of the relevant national and institutional committees on human experimentation and with the Helsinki Declaration of 1975, as revised in 2008.

### Participants

The sample consisted of three groups of participants: psychiatric patients with persistent AVHs, participants from the general community who experienced persistent AVHs, and healthy controls without AVH. Part of this sample was shared with our previous studies^45^,^46^, although analysis in relation to the current research questions has not been previously reported. All participants provided informed written consent for study participation.

Clinical participants were recruited from the University Medical Centre Utrecht. Patients who received routine treatment for psychosis or second opinion on refractory psychosis in our clinic were invited to join the study. Inclusion criteria were as follows: age 18–65 years, and regular experience of AVHs for over a year. The Comprehensive Assessment of symptoms and History (CASH)^47^ was conducted by an independent psychiatrist to confirm psychiatric diagnoses and assess alcohol and substance use.

Non-clinical voice hearers were included in the study if they were 18–65 years old, experienced AVHs at least once a month, and did not meet criteria for any diagnosis on the Diagnostic and Statistical Manual of Mental Disorders, Fourth Edition (DSM-IV)^48^ nor for a personality disorder as per SCID-II diagnosis. Healthy controls were included if they were 18–65 years old, reported absence of AVH experience, and did not meet criteria for any DSM-IV (axis 1 or 2) diagnosis. For all three groups, participants were excluded if they had alcohol or substance dependence.

The two non-clinical groups were recruited and identified according to the following steps. Firstly, individuals who visited a Dutch mental health website “Explore Your mind” (www.verkenuwgeest.nl) were invited to fill out a self-test on AVH. This self-test was based on the Launay and Slade Hallucinations Scale (LSHS)^49^. Individuals who scored a total of 7 or above on two LSHS items (“In the past, I have had the experience of hearing a person’s voice and then found that no one was there” and “I have been troubled by voices in my head”), and those who scored 0 on both items, were identified for further screening by trained psychologists. In the second stage, individuals (both high and low LSHS scorers) were interviewed over the phone to see if they fulfilled the following criteria: (1) no diagnosis or treatment for psychiatric disorders other than depressive or anxiety disorders in complete remission; (2) no alcohol or substance abuse for at least 3 months; and (3) no chronic somatic disorder. Individuals who scored high on the two LSHS items were further inquired whether (4) their voices were distinct from thoughts and had a perceptual quality; and that (5) their voices were experienced at least once a month for over 1 year. At the third stage of selection, participants who fulfilled all the above criteria were invited to attend a clinical interview, which consisted of the CASH interview^47^ and the Structured Clinical Interview for Personality Disorder (SCID-II)^50^. Non-clinical individuals were screened for alcohol and substance dependence by self-report, followed by urine tests. Ten non-clinical individuals were declined participation in this study due to positive screening result of alcohol or substance dependence.

### Measures

#### The revised Launay-Slade Hallucination Scale (LSHS)

To screen for hallucinations in the non-clinical groups, the present study adopted a modified, 17-item version of the LSHS^49^.

#### Psychotic Symptom Rating Scales (PSYRATS)

Aspects of hallucinatory experiences were assessed using the 11-item auditory hallucinations scale of the PSYRATS interview, which has been used in clinical and non-clinical voice hearers^51^,^52^. The two items on negative content of voices (items 6 “Degree of negative content” and 7 “Amount of negative content”) and the two distress items (items 8 “Amount of distress” and 9 “Intensity of distress”) were averaged into two combined measures respectively due to high correlations between the component items (Negative content: *r* = 0.766, *p* < 0.001; Distress: *r* = 0.787, *p* < 0.001).

#### Beliefs about Voices Questionnaire-Revised (BAVQ-R)

Beliefs about voices and emotional and behavioral response to voices were assessed using the BAVQ-R. Good internal consistency and test-retest reliability of the scale have been reported in clinical samples^53^ and non-clinical voice hearers^54^.

#### The revised NEO Personality Inventory (NEO–PI–R)

The revised NEO Personality Inventory is a 240-item questionnaire that assesses five key personality dimensions, including Neuroticism, Extraversion, Openness to Experience, Agreeableness and Conscientiousness^1^. Only the Neuroticism subscale was reported in the present study. The Cronbach’s α for NEO neuroticism score was previously reported as 0.93 among non-clinical participants, and 0.91 among patients with substance dependence^55^,^56^.

#### Child Trauma Questionnaire–Short Form (CTQ-SF)

Childhood trauma was assessed using a 25-item CTQ-SF. Frequency of five types of trauma was assessed–namely sexual abuse, physical abuse, emotional abuse, physical neglect and emotional neglect. Good internal consistency reliability has been reported in both clinical^57^ and community samples^58^.

### Statistical analysis

Group differences were tested by using one-way MANOVAs and ANOVAs. In all group comparison analyses, gender, age and years of education were entered as covariates. In view of the unbalanced group sizes, we conducted bootstrap processes (resampling for 1000 times) to check the robustness of group differences.

To examine associations between neuroticism and cognitive-affective reactions to AVH (distress, resistance, perceived malevolence, and perceived power), hierarchical linear regression models were built, with the respective cognitive-affective variable as dependent variable (DV). Gender (dummy recoded), age and years of education as independent variables (as IVs) were entered at the first level (where they were significant predictors), neuroticism and group (dummy recoded) as IVs entered at the second level, and interaction term neuroticism (grand-centred) x group as IV at the third level.

To examine the mediation effect of neuroticism on the relationships between childhood trauma and the cognitive-affective responses to AVH, we adopted the causal steps approach developed by Baron and Kenny^59^. In each mediation model, CTQ-SF total score was entered as IV, neuroticism as mediator, and each of the following variables as respective DV (distress, resistance, perceived malevolence, and perceived power).

Analyses were performed on SPSS 22. Missing values were handled by case-wise deletion.

## Results

The final sample consisted of 40 patients with a psychiatric diagnosis, 135 non-clinical voice hearers and 126 healthy individuals.

### Demographic and clinical variables

As shown in Table 1, the three groups were matched on age and gender ratio. Healthy individuals completed more education than both voice-hearing groups (*p*s < 0.05).

All patients had a diagnosis of schizophrenia spectrum or other psychotic disorder: schizophrenia (*n* = 4), schizoaffective disorder (*n* = 1), and psychotic disorder NOS (*n* = 35). Some of them had additional (concurrent or past) diagnoses as follows: bipolar I or II disorder (*n* = 4), major depressive disorder (*n* = 4), personality disorder (*n* = 14), dissociative disorder NOS (*n* = 2), and religious or spiritual problem (*n* = 1).

Table 2 displays the characteristics of AVH reported by patients and non-clinical voice hearers on PSYRATS. One-way MANOVA revealed a significant group difference on PSYRATS scores (Wilks’ Lambda *F*(9,125) = 8.29, *p* < 0.001, η^2^_p_ = 0.37), after adjusting for demographic variables. Follow-up univariate ANOVAs revealed that patients scored significantly higher than non-clinical voice hearers on the following PSYRATS dimensions: duration, negative content, distress and disruption to life (*p*s < 0.006, threshold Bonferroni corrected). Results of group differences remained the same before and after bootstrapping.

Table 2 displays appraisals of AVH reported by the two voice hearer groups. One-way MANOVA revealed a significant group difference on BAVQ-R scores (Wilks’ Lambda *F*(5, 131) = 4.47, *p* < 0.001, η^2^_p_ = 0.15), after adjusting for demographic variables. Follow-up univariate ANOVAs revealed that patients scored significantly higher than non-clinical voice hearers on resistance, malevolence and power, while significantly lower on engagement and benevolence (*p*s < 0.01, threshold Bonferroni corrected). Results of group differences remained the same before and after bootstrapping.

### Level of neuroticism across groups

One-way ANOVA revealed a significant group difference in neuroticism score on NEO-PI-R (*F*(2,287) = 10.08, *p* < 0.001, η^2^_p_ = 0.07) after adjusting for demographic variables. Post-hoc Bonferroni tests revealed that neuroticism score was higher in patients (*M* = 3.07, *SD* = 0.24) than non-clinical voice hearers (*M* = 2.96, *SD* = 0.21; *SE* = 0.04, *p* = 0.020), and higher in non-clinical voice hearers than healthy controls (*M* = 2.90, *SD* = 0.17; *SE* = 0.03, *p* = 0.030). Bootstrapping yielded a consistent result of group comparisons.

### Association between neuroticism and cognitive-affective responses to voices

Hierarchical linear regression models revealed that hallucinatory distress was significantly associated with age (β = −0.20, *t* = −2.42, *p* = 0.017), but not with gender or years of education (*p*s > 0.05). With the effect of age adjusted for, neuroticism and group significantly predicted the amount of hallucinatory distress (β = 0.18, *t* = 2.29, *p* = 0.024 and β = 0.32, *t* = 4.15, *p* < 0.001, respectively; see Table 3). The neuroticism x group interaction effect was non-significant (*p* = 0.748), suggesting that strength of the association between neuroticism and hallucinatory distress did not differ between clinical and non-clinical voice hearers.

Hierarchical linear regression models revealed a significant age effect on resistance (β = −0.21, *t* = −2.51, *p* = 0.013) and malevolence (β = −0.21, *t* = −2.44, *p* = 0.016). After adjusting for age, both resistance and malevolence were significantly predicted by neuroticism (β = 0.27, *t* = 3.47, *p* = 0.001 and β = 0.16, *t* = 2.04, *p* = 0.044, respectively) and group (β = 0.27, *t* = 3.51, *p* = 0.001 and β = 0.27, *t* = 3.47, *p* = 0.001, respectively; see Table 3). The neuroticism x group interaction effect was not significant for either resistance or malevolence (*p*s > 0.05), indicating that the associations between neuroticism and resistance against AVH and perceived malevolence of voices were not significantly different between clinical and non-clinical voice hearers.

Hierarchical linear regression models revealed no significant effect of age, gender or years of education on power. Neuroticism and group significantly predicted power (β = 0.19, *t* = 2.28, *p* = 0.024 and β = 0.22, *t* = 2.66, *p* = 0.009, respectively), whereas their interaction effect did not reach significance (β = 0.06, *t* = 0.58, *p* = 0.565; see Table 3).

As an exploratory investigation, mediation models were tested, with neuroticism as IV, perceived malevolence and power of voices as mediators, and hallucinatory distress and resistance as DVs. Malevolence and power significantly mediated the association between levels of neuroticism and distress (Sobel tests: for malevolence *z* = 3.16, *p* = 0.002; for power *z* = 2.76, *p* = 0.006), and the association between levels of neuroticism and resistance (Sobel test: for malevolence *z* = 3.16, *p* = 0.002; for power *z* = 2.74, *p* = 0.006).

### Relationship between childhood trauma, neuroticism and cognitive-affective reactions to AVH

One-way MANOVA showed a significant group difference on CTQ-SF scores (Wilks’ Lambda *F*(10,562) = 6.18, *p* < 0.001, η^2^_p_ = 0.10), after adjusting for demographic variables (see Table 4). Follow-up univariate ANOVAs revealed significant group differences on CTQ-SF total score and all subscales (*p*s ≤ 0.001). Patients had higher scores than non-clinical voice hearers on physical abuse and the total score (*p*s < 0.05), while both AVH groups had higher scores on all CTQ-SF dimensions than the healthy controls (*p*s < 0.01). Results of group differences remained the same before and after bootstrapping.

Mediation models of neuroticism, childhood trauma, and cognitive-affective reactions to AVH by using data from both clinical and non-clinical voice hearers (*n* = 175) are shown in Table 5. After adjusting for the effect of age, CTQ-SF total score significantly predicted distress, resistance, malevolence, and power (Step 1). However, for all models, when neuroticism was entered into the regression (i.e. Step 2), the predictive effect of CTQ-SF total score reduced to non-significance and that of neuroticism becomes significant. These results suggested that neuroticism fully mediated the relationship between childhood trauma and cognitive-affective reactions to AVH.

To further explore the effects of neuroticism on mediating the links between specific types of childhood trauma and reactions to AVH, additional mediation analyses were conducted (test statistics available from the first author). Rather than testing the mediation effect of CTQ-SF total score as a whole, we tested the mediation effects of each of the CTQ-SF subscores respectively. At Step 1, we found that emotional abuse and physical abuse significantly predicted distress, resistance, malevolence, and power. Emotional neglect predicted distress and resistance, whereas physical neglect predicted resistance and power. Sexual abuse did not predict any of the cognitive-affective reactions to AVH. At Step 2, the effects of emotional abuse and physical abuse on all four aspects of cognitive-affective reactions to AVH were fully mediated by neuroticism. The effects of emotional neglect and physical neglect on resistance were fully mediated by neuroticism. The effect of emotional neglect on distress and the effect of physical neglect on power were partially mediated by neuroticism.

## Discussion

This study examined the relationship between neuroticism, cognitive-affective reactions to auditory verbal hallucinations (AVHs), and childhood trauma. In view of the evidence that AVHs are experienced by patients with varied psychiatric diagnoses, as well as individuals in the general population, the present study adopted a single-symptom approach and compared patients experiencing frequent AVHs, non-patients experiencing frequent AVHs, and non-voice-hearers in the general population. All our hypotheses were confirmed: (i) we found a positive association between neuroticism and level of hallucinatory distress; (ii) there was a positive association between neuroticism and a tendency to resist AVHs and to appraise them as malevolent and powerful; and (iii) neuroticism mediated the relationship between childhood traumatic experiences and cognitive-affective reactions to AVHs.

We demonstrated the role of neuroticism in predicting cognitive-affective responses to AVHs. Whilst there was a graded difference in level of neuroticism across the three groups, it is most intriguing that, in both clinical and non-clinical voice-hearers alike, a higher level of neuroticism was associated with more emotional distress and behavioral resistance in response to AVHs, as well as a stronger tendency to perceive voices as malevolent and powerful. Therefore, even among voice hearers who did not have a diagnosis of any psychotic disorder, those who are higher on neuroticism are more likely to respond to voices in a way that is characteristic among patients with psychosis, albeit to an attenuated degree. Consistent with the stress research findings that neuroticism heightens an individual’s affective reactivity to stressors and renders an individual to consider experiences as dangerous and threatening^26^,^27^, our results support that such links between neuroticism and cognitive-affective reactions to general stressors applies also to the psychopathology of AVH. More specifically, we found that the association between neuroticism and hallucinatory distress was fully mediated by perceived malevolence of voices. To put into the cognitive-behavioral framework of hallucinations^60^,^61^,^62^, it is possible that voice-hearers who are more neurotic are more emotionally reactive to voices and appraise voices as more negative and powerful, hence adopting a resistant and avoidant coping strategy, which in turn maintains the severity and distress of the voices^11^,^12^.

The present study added to the literature on the relationship between childhood trauma and hallucinations. We found that childhood trauma was related to how an individual responds to the voices cognitively and affectively. Such association was the most robust for emotional abuse and physical abuse, and was also evident for emotional neglect and physical neglect. Notably, all effects of childhood trauma on psychological reactions to AVH were mediated by neuroticism. Therefore, even though it has been suggested that a history of trauma does not necessarily affect the negativity of voice content^45^, it contributes to overall occurrence of AVHs, and more specifically, maladaptive reactions to voices by increasing the individual’s emotional reactivity and sense of threat. As this was the first time that childhood trauma was found to predict cognitive-affective reactions to AVHs via neuroticism, our mediation results warrant replication by future studies.

There are several limitations to this study. First of all, this study set out to recruit patients with experiences of AVHs, without limiting to psychotic disorders only. Therefore, whilst all patients had a psychotic disorder, some presented with comorbidities. There was also heterogeneity in terms of symptom profile, duration of illness, and response to treatment. It has been shown that the experience of and reactions to AVH may differ across stages of illness^63^,^64^ and diagnosis^65^,^66^,^67^. Although this sample was representative of the recruitment site, we cannot quantify how the link between neuroticism and the individual’s reactions to voices compares between patients with or without comorbidities, or patients at different stages of illness. Therefore, one needs to be cautious when generalizing our findings to more specific diagnostic groups. Secondly, although we have excluded non-clinical participants with active emotional disorders, since neuroticism has been shown to be associated with levels of depression and anxiety, including a clinical measure of these emotions would have made our methodology more stringent. Thirdly, the reliability of retrospective self-report of childhood trauma may be questionable. Against these caveats, this study extended the current literature on auditory verbal hallucinations by examining the associations between individuals’ personality, cognitive and affective reactions to voices, and history of trauma.

In conclusion, our results shed more light on how voice hearers interact with the symptom, and the role of neuroticism as a putative mechanism linking childhood trauma and psychological reactions to voices.

## Additional Information

**How to cite this article**: So, S. H.-w. *et al*. Relationship between neuroticism, childhood trauma and cognitive-affective responses to auditory verbal hallucinations. *Sci. Rep.* **6**, 34401; doi: 10.1038/srep34401 (2016).

## Figures and Tables

**Table 1:** Demographic information across groups.

	Patients (*N* = 40)	Non-clinical voice hearers (*N* = 135)	Healthy controls (*N* = 126)	Group comparison
Age	45.43 ± 11.95	50.60 ± 12.70	50.80 ± 14.52	*F*(2)* = *2.69, *p = 0.*070, η^2^_p_ = 0.02
Gender	32F^a^ (80.0%)	92F (68.1%)	86F (68.3%)	*x*^2^(2) = 2.29, *p* = 0.318, Cramer’s *V* = 0.09
Years of Education	13.15 ± 2.90	13.35 ± 2.12	14.05 ± 2.30	*F*(2)* = *3.81, *p = 0.*023, η^2^_p_ = 0.03

^a^F means female.

**Table 2:** Characteristics of and responses to AVH (*M* ± *SD*) on PSYRATS and BAVQ-R across groups.

	Patients (*N* = 40) *M* ± *SD*	Non-clinical voice hearers (*N* = 135) *M* ± *SD*	*F*-test
PSYRATS scores			
Frequency	1.92 ± 1.24	1.54 ± 1.22	*F* = 3.77, *p* = 0.05
Duration	2.15 ± 1.01	1.54 ± 0.73	***F***** = 16.98,** ***p***** < 0.001**
Location	2.21 ± 1.11	2.21 ± 1.15	*F* = 0.03, *p* = 0.86
Loudness	1.70 ± 0.72	1.80 ± 0.67	*F* = 0.80, *p* = 0.37
Belief of origin	2.68 ± 1.19	3.16 ± 1.13	*F* = 6.14, *p* = 0.01
Negative content	1.74 ± 1.36	0.56 ± 1.03	***F***** = 35.91,** ***p***** < 0.001**
Distress	1.50 ± 1.25	0.53 ± 0.98	***F***** = 26.02,** ***p***** < 0.001**
Disruption to life	1.20 ± 1.29	0.23 ± 0.61	***F***** = 49.37,** ***p***** < 0.001**
Controllability	2.38 ± 1.48	1.82 ± 1.49	*F* = 3.51, *p* = 0.06
BAVQ-R scores			
Malevolence	5.28 ± 5.86	1.74 ± 3.83	***F***** = 16.02,** ***p***** < 0.001**
Benevolence	7.20 ± 5.29	10.20 ± 4.51	***F***** = 8.37,** ***p***** = 0.004**
Power	6.88 ± 4.92	4.75 ± 2.93	***F***** = 10.69,** ***p***** = 0.001**
Engagement	8.65 ± 7.98	12.34 ± 7.08	*F* = 4.75, *p* = 0.03
Resistance	10.58 ± 8.56	4.50 ± 6.76	***F***** = 20.66,** ***p***** < 0.001**

“Negative content” score was the average of PSYRATS items 6 (amount of negative content) and 7 (degree of negative content), whereas “Distress” score was the average of PSYRATS items 8 (amount of distress) and 9 (intensity of distress). Significant findings are indicated in bold.

**Table 3:** Hierarchical regression analyses for testing the association between neuroticism and cognitive-affective responses to AVH.

	Distress	Resistance	Malevolence	Power
Coefficients				
1. β(Age)	**−0.20**	**−0.21**	**−0.21**	—
2. β(Neuroticism)	**0.18**	**0.27**	**0.16**	**0.19**
β(Group)	**0.32**	**0.27**	**0.27**	**0.22**
3. β(Neuroticism × Group)	−0.03	−0.02	−0.02	0.06
Equation *F*	**11.92**	**13.53**	**9.88**	**7.91**
*df*	3,142	3,143	3,143	2,144
*R*^2^	0.20	0.22	0.17	0.10

Significant findings are indicated in bold. In the regression model for each dependent variable, age was entered at the first level (except for in the model for power), neuroticism and group at the second level, and neuroticism × group at the third level of independent variables. As the neuroticism × group interaction was not significant, Equation *F*, *df,* and *R*^2^ were based on the models with the interaction terms excluded.

**Table 4:** Childhood traumatic experiences (*M* ± *SD*) on CTQ-SF across groups.

	Patients (*N* = 40)	Non-clinical voice hearers (*N* = 135)	Healthy controls (*N* = 126)	*F*-test (*F*, *p*)
Emotional abuse	13.23 ± 6.73	10.67 ± 4.96	7.23 ± 2.86	***F***** = 27.85,** ***p***** < 0.001**
Physical abuse	8.13 ± 5.05	6.55 ± 3.33	5.47 ± 1.25	***F***** = 10.31,** ***p***** < 0.001**
Sexual abuse	8.73 ± 5.69	7.66 ± 4.48	5.73 ± 1.85	***F***** = 9.56,** ***p***** < 0.001**
Emotional neglect	15.10 ± 5.55	12.81 ± 4.97	10.86 ± 4.18	***F***** = 9.81,** ***p***** < 0.001**
Physical neglect	8.53 ± 3.86	7.50 ± 2.65	6.62 ± 2.07	***F***** = 7.18,** ***p***** = 0.001**
Total score	53.70 ± 21.84	45.19 ± 15.61	35.87 ± 8.50	***F***** = 22.04,** ***p***** < 0.001**

Significant findings are indicated in bold.

**Table 5:** Regression analyses for testing the mediation effects of neuroticism in the relationship between childhood trauma and cognitive-affective reactions to AVH.

	Distress		Resistance		Malevolence		Power	
	Step 1	Step 2	Step 1	Step 2	Step 1	Step 2	Step 1	Step 2
Coefficients								
1. β(Age)	**−0.23**	**−0.19**	**−0.25**	**−0.18**	**−0.25**	**−0.21**	**−**0.15	**−**0.11
β(CTQ)	**0.18**	0.14	**0.19**	0.12	**0.17**	0.13	**0.18**	0.14
2. β(Neuroticism)		**0.21**		**0.29**		**0.19**		**0.18**
Equation *F*	**6.41**	**6.57**	**7.16**	**9.61**	**6.69**	**6.26**	**3.87**	**4.19**
*df*	2,143	3,142	2,144	3,143	2,144	3,143	2,144	3,143
*R*^2^	0.07	0.10	0.08	0.15	0.08	0.10	0.04	0.06
Δ*R*^2^	0.08	0.04	0.09	0.08	0.09	0.03	0.05	0.03
*F*_change_	**6.41**	**6.40**	**7.16**	**13.29**	**6.69**	**5.04**	**3.87**	**4.65**

Significant findings are indicated in bold. In each Step1, age and CTQ were entered as independent variables; and in Step 2, neuroticism was entered as a third independent variable.
